# Concordance of Mother-Child (6–23 Months) Dietary Diversity and Its Associated Factors in Kucha District, Gamo Zone, Southern Ethiopia: A Community-Based Cross-Sectional Study

**DOI:** 10.1155/2021/8819846

**Published:** 2021-10-14

**Authors:** Tesfaye Guja, Yabsira Melaku, Eshetu Andarge

**Affiliations:** ^1^Department of Public Health, Arba Minch College of Health Sciences, P.O. Box 155, Arba Minch, Ethiopia; ^2^Department of Nutrition and Dietetics, Faculty of Public Health, Institute of Health Sciences, Jimma University, P.O. Box 378, Jimma, Ethiopia; ^3^School of Public Health, College of Medicine and Health Sciences, Arba Minch University, P.O. Box 021, Arba Minch, Ethiopia

## Abstract

Meeting minimum standards of dietary quality in mothers and children is a challenge in many developing countries including Ethiopia. Emerging evidence suggests that maternal and child dietary diversity is associated, but little is known about the associated factors of concordance of mother-child dietary diversity in Ethiopia and none is documented in the study area. This study examines the concordance between mother-child (6–23 months) dyads dietary diversity and the associated factors in Kucha District, Gamo Zone, Southern Ethiopia. A community-based cross-sectional study was conducted among 791 mother-child (6–23 months) pairs from 11 selected kebeles on March 6 to April 13, 2017. Multistage cluster sampling technique was used to select the study subjects. The sampling frame was obtained from the family folder of health posts in each kebele. The mother-child pairs were selected by the simple random sampling method. The 7 food groups of the World Health Organization (WHO) for children and the 10 food groups of FANTA/FAO 2016 for mothers were used to analyze the dietary diversity. Cohen's kappa statistics was calculated to see the strength of concordance. The multivariable logistic regression model was fitted to determine factors affecting mother-child dietary diversity concordance. A good concordance was noted between mother-child dietary diversity scores (Kappa = 0.43). Only 56 (7.1%) mothers were negative deviants, and 133 (16.8%) mothers were positive deviants in dietary diversity consumption. Rural residence (AOR = 3.49; 95% CI: 1.90–6.41), having no formal education (AOR = 1.8; 95% CI: 1.08–3.05), not owning milking cow (AOR = 1.7; 95% CI: 1.10–2.56), children with low dietary diversity (AOR = 8.23; 95% CI: 5.17–13.08), and mothers with low dietary diversity (AOR = 0.46; 95% CI: 0.29–0.74) were found to be factors associated with mother-child dietary diversity concordance. An increase in the percentage of children reaching the minimum dietary diversity was greater with a successive increase in maternal dietary diversity. Despite interesting similarities between mothers and children dietary consumption, more than three-quarters of concordants did not achieve the recommended dietary diversity score (were low concordants). Interventions targeting on rural women's access to high school education, home-based milking cow rearing, and promoting nutrition-sensitive agriculture to meet the dietary requirements of mothers and children in a sustainable manner and public health efforts to improve child nutrition may be strengthened by promoting maternal dietary diversity due to its potential effect on the entire family.

## 1. Background

Maternal and child undernutrition and micronutrient deficiency affect approximately half of the world's population [1]. Micronutrient malnutrition is a widespread nutrition challenge faced by women living in resource-poor settings, the consequences of which affect not only the health and survival of women but also that of their children. One of the main factors responsible for this type of malnutrition is the poor quality of women's diets as they lack dietary diversity [2]. Greater than two-thirds of malnutrition-related child deaths are associated with inappropriate feeding practices during the first two years of life in such a way that infants and young children received inadequately nutritious diets and poorly diversified [3]. Adequate feeding interventions alone were estimated to prevent almost one-fifth of under five-year children mortality in developing countries [4]. Adequate nutrition is fundamental to proper growth and development of children and for survival as well as for health and reproductive performance of women [5]. Therefore, providing sustainable diets rich in micronutrients and macronutrients is vital in the effort to combat malnutrition in mothers and children [6].

Dietary diversity (DD)—the sum of food groups consumed over a period of 24 hours—has been documented as a valid and reliable indicator of dietary quality and nutrient adequacy. This has been explained by the fact that there is no any single food that contains all the required nutrients for optimal health [7, 8]. Moreover, promotion of diverse diet is one of the several approaches to improving micronutrient nutrition for women of reproductive age [2]. Because of the perceived importance of dietary diversity for health and nutrition, indicators of dietary diversity have become increasingly popular in recent years [9]. Dietary diversity score (DDS) is a reasonably easy-to-measure proxy variable for young children's nutrient intake, and the World Health Organization (WHO) uses dietary diversity as one of the key indicators to assess child feeding practices [10, 11]; individuals consuming more diverse diets are thought to be more likely to meet their nutrient needs [9]. Dietary diversity is also a proxy indicator of diet quality for women of reproductive age in resource-poor settings [12]. In developed countries the diets of lactating mothers reflect not only their own intake but also the diets of their small children and families as well [7, 13]. That is, maternal dietary diversity is also strongly linked to that of infants in the same household and to the average household nutrient adequacy. In short, lactating mothers with higher dietary diversity have children and family with higher dietary diversity [7]. A recent study of mothers and children found that if mothers had poor diet, their infants were at increased risk for poor diet quality [14, 15]. Dietary diversity is an important component of dietary quality: consumption of a higher number of food items and food groups is associated with improved nutritional status [16].

Meeting minimum standards of dietary quality is a challenge in resource-scarce countries; though a number of successful strategies have been developed to improve feeding practices in under two-year children. In such situations, household food security is poor, diets are based predominantly on starchy staples and seasonal fruits and vegetables, and it has often not been given enough emphasis [9, 17–19]. Owing to the high demand on energy and nutrients for vulnerable infants and young children, the problem is particularly critical to them [9]. It is a vicious cycle: generation after generation, children are robbed of their potential because they cannot get access to good nutrition. Those lost futures take an enormous toll on the country's economic well-being. Hence, ensuring maternal dietary diversity to the acceptable level is very important, which in turn may enhance the dietary diversity of children that will help in tackling maternal and child malnutrition. Ethiopia recognizes that it must attack the problem on many fronts, including improving agriculture, food quality, and micronutrient fortification [20]. However, a cross-sectional study conducted in Gamo Gofa Zone, South Ethiopia, showed that 76.7% of under 2-year children fed ≤3 food items within 24 hours preceding the survey, which is below the minimum standard of dietary diversity score [21].

To our knowledge, there is a dearth of evidence on dietary patterns and diet quality for women and young children in Ethiopia. Much of the available information focused on pregnant women, while lactating women are also vulnerable [22]. Evidence in developing countries disclosed that diets of mothers and children vary owing to the manifold factors arising from cultures and beliefs within the community [23–27]. A study based on the 2008 Ghana Demographic and Health Survey revealed a significant positive association between child and maternal DDS [28]. Such studies investigating the association between maternal and child dietary diversity and its predictors in Ethiopia are limited. Hence, there is a need to determine the concordance between mother and child dietary diversity in order to design culturally appropriate, cost-effective, and evidence-based programs against the prevailing malnutrition in the country.

## 2. Methods

### 2.1. Study Setting and Design

The study was conducted in Kucha District located 450 km away from the country's capital, Addis Ababa, and 215 km from the regional capital, Hawassa. The district is located in Gamo Zone in the Southern Nations, Nationalities, and Peoples' Region (SNNPR) and contains a total of 35 (32 rural, 3 urban) administrative subdistricts (kebeles). According to the Kucha District Health Office estimate, the district has a total population of 189,233 in 2017 and of which mothers 15–49 years were 37,544 (19.8%) and 6,642 (3.5%) were children (6–23 months). The district has eight health centers, thirty-nine health posts, one preparatory school, eight high schools, and fifty-one second-cycle and eighteen first-cycle primary schools [29]. A community-based cross-sectional study was conducted from March to April, 2017, among mother-child pairs (both breastfeeding or not with children aged 6–23 months) who were permanent residents of the district and able to provide information (free of mental illness and communication difficulties).

### 2.2. Sample Size Determination and Sampling Technique

The sample size was determined by using the single population proportion formula with the assumptions of 95% confidence level, estimated proportion of discordance in mother-child DD of 50%, 5% margin of error, and the minimum sample size (*n*_o_) of 384. Since the source population was 6642 that is less than 10,000, we have reduced the sample to 362 by using the finite population correction formula. By considering (10%) nonresponse rate and a design effect of 2, the final sample size was 796.

From the total 35 kebeles of the district, eleven kebeles were selected using simple random sampling/SRS/lottery method. To identify mother-child (6–23 months) pairs from the selected kebeles, the family folder (registry book of all families with their children) within the health post was used. Using these registered data as a sampling frame in each kebele, the required number of samples was determined for each kebele with consideration of size of mother-child pairs in each kebele. The required numbers of women interviewed in each kebele were selected randomly from the sampling frame using the “select random samples” command in the Statistical Package for Social Sciences (SPSS) software. In case of twins, one of the twins was randomly selected. When two or more children in the specified age range were present in one HH, the last child with his/her mother was selected.

### 2.3. Data Collection Methods and Measurements

Data were collected by ten nurses who were recruited as data collectors and supervised by two BSc nurses. Mothers were asked to respond on the diet they fed their children and their own feeding in the past 24 hours and their sociodemographic characteristics. They were asked to recall all foods and beverages the child fed during the past 24 hours, both within and outside the home. A semistructured pretested questionnaire was used to collect data on variables pertaining to sociodemographic characteristics as well as dietary, health care practices, and other related variables of mothers and their children (6–23 months old). The questionnaire was first developed in English, translated to the local language *Gamotho*, and then back translated to English by an independent translator for consistency.

Minimum dietary diversity of children: proportion of children 6–23 months of age who receive foods from ≥4 food groups during the previous day considered adequate and <4 food groups is considered inadequate (low) from the seven defined food groups the previous day and night. A cutoff point of 4 was used to assess the adequacy of a child's DDS; hence, a child with DDS ≥ 4 was considered to have a high diet diversity (adequate diet) and otherwise DDS < 4 was considered a child with low diet diversity (inadequate diet) [30].

Minimum dietary diversity of women (MDD-W): a cutoff point of 5 food groups was used to assess the adequacy of a mother's DDS; hence, a mother with DDS ≥ 5 was considered to have a high dietary diversity (adequate diet) and otherwise DDS < 5 considered a mother with low diet diversity (inadequate diet) [16]. The proportion of mothers who reach this minimum in a population is used as a proxy indicator for higher micronutrient adequacy, one important dimension of diet quality [16, 31].

Household food insecurity was assessed using the Household Food Insecurity Access Scale (HFIAS) developed by FANTA, and food security status was classified into four categories: food secured, mild, moderate, and severely food insecure. It records household reactions and response to food access problems faced during a recall period of four weeks. It aims to capture the severity of food insecurity faced by households due to lack of or limited resources to access food. The respondent is first asked an occurrence question, that is, whether the condition in the question happened at all in the past four weeks (yes or no). If the respondent answers “yes” to an occurrence question, a frequency-of-occurrence question is asked to determine whether the condition happened rarely (once or twice), sometimes (three to ten times), or often (more than ten times) in the past four weeks [32].

Wealth index: to measure the wealth index, a wealth index measurement tool adapted from EDHS was used [33]. It was classified using terciles (low, medium, high).

Concordance: agreement of dietary diversity in mother-child dyads. If the mothers eat ≥5 food groups from the ten food groups and her child eats ≥4 foods from the seven food groups (high concordant who achieved the recommended minimum dietary diversity), or when the mothers eat <5 food types from the ten food groups and her child eats <4 foods groups from the seven food groups (low concordant who did not achieve minimum dietary diversity) in the previous day (24 hours) of the survey was termed as mother-child dietary diversity concordance.

Discordance: disagreement on dietary diversity consumption between mothers and children. Mothers >5 and children <4 food groups or mothers <5 food groups and children >4 food groups.

Negative deviant: among the discordant mothers who ate ≥5 food groups from ten food groups of MDD-W (meeting high dietary diversity criteria of the FANTA and FAO) but who fed their children <4 food groups (not meeting minimum dietary diversity, WHO criteria).

Positive deviant: among the discordant mothers who eat <5 food groups from ten food groups of MDD-W (low dietary diversity) but who fed their children ≥4 food groups (meeting the WHO criteria of minimum dietary diversity of children).

High concordant: mothers/children who achieved the minimum dietary diversity and being concordant.

Low concordant: mothers/children who did not achieve the minimum dietary diversity and being concordant.

Dietary diversity level: considered high if the DDS is ≥4 in children and ≥5 in mothers; otherwise considered low.

### 2.4. Data Quality Assurance

.The questionnaire was pretested on 5% of the sample of mother-child pairs in the Boreda district out of the study area, and the necessary changes were made to it before data collection. Two-day training was given on the aim of the research, content of the questionnaire, and the interview process for data collectors and supervisors to increase their performance in the activities. Data were collected on all days of the week since people may eat differently on different days of the week. The collected data were checked every day by supervisors and the principal investigator for its completeness and consistency. All the interviews were conducted at the residences of the study participants. Vacant or closed houses during the day of visit were revisited two times to maintain the required sample size. Probing technique was used in 24-hour dietary data to minimize recall bias.

### 2.5. Data Analysis

After checking the data for completeness and missing values, they were coded and entered using EpiData, version 3.1, cleaned and analyzed using SPSS statistical software version 20.0. Descriptive statistics for categorical variables was presented as frequency percent, and continuous variables were presented using mean ± SD and percentage and to examine the differences among low and high dietary diversity of mothers and children. Principal component analysis was done to set household wealth score; the score was ranked into terciles (low, middle, and high). The HFIAS score was calculated for each household food insecurity status by summing the codes for each frequency of occurrence of the condition questionnaire. The score for a household ranges from 0 to 27, with a maximum score of 27 indicating most food-insecure households and ranked into secure, mildly insecure, moderately insecure, and severely insecure. Finally, food insecurity was categorized as secure and insecure (mild, moderate, and severe). Bivariate analysis was done to examine the associations between the concordance of maternal-child dietary diversity and each of the independent variables independently. To identify the predictors of maternal-child dietary diversity concordance, variables that were significantly associated at *P* value (<0.25) in the bivariate analysis were entered into the multivariable logistic regression model. Those variables with *P* value < 0.05 in the multivariable analysis were declared as significant. Adjusted odds ratios (AOR) with 95% confidence level showed the strength of association between the predictors and the dependent variable. The Hosmer–Lemeshow test was checked for model fitness. Cohen's Kappa value was calculated to measure the strength of concordance between the dietary diversity score categories calculated for mothers and children.

### 2.6. Ethics Approval and Consent to Participate

Ethical clearance was obtained from Institutional Research Review Board, Institute of Health, Jimma University. Written permission was obtained from the Gamo Zone (the then Gamo Gofa Zone) Health Department and Kucha District Health Office. During data collection, informed written consent was obtained from women who participated in the study. Confidentiality of mothers' and children's information was maintained during data collection, analysis, and interpretation.

## 3. Results

### 3.1. Sociodemographic and Economic Characteristics of the Mothers

A total of 791 pairs of mothers (15–49 years) and children (6–23 months) participated in the study, making the response rate 99.4%. About half 389 (49.2%) of mothers were between the age of 25 and 34, and the mean age was 27.38 years ± 5.36 (SD). Moreover, the vast majority 720 (91%) of mothers reported that they were married. With regard to education, less than half 362 (45.9%) reported that they had no formal education. Majority 718 (90.8%) of mother-child pairs were rural dwellers, and about half 406 (51.3%) had four or more family members in their households. In respect to maternal status in the household, majority 709 (89.6%) were from male-headed households and about three-fourths 589 (74.5%) obtain food for consumption from their own production (farming), and only 96 (12.1%) of mothers grow vegetables in their backyards. Regarding diversified diet consumption, most 554 (70%) had received dietary advice/information either from health professionals, or mass media, or their families. About one-quarter 195 (24.7%) of the mothers and one-third 272 (34.4%) of the children consumed the recommended minimum dietary diversity. A slightly more than one-third of the households 312 (39.4%) were food secure; and 257 (32.5%) were from households with high wealth (rich family) (Table 1).

### 3.2. Dietary Consumption Pattern of Mothers and Children

In the study, both mothers and children almost universally consumed grains, roots, and tubers. Almost all mothers 786 (99.4%) and the vast majority of children 758 (95.8%) consumed these foods in the preceding day of the study. A very small percentage 36 (4.6%) of mothers and 36 (4.6%) of children consumed flesh foods. Only 70 (8.8%) mothers and 148 (18.7%) children consumed eggs. More than two-thirds 568 (71.8%) of children and less than one-quarter 159 (20.1%) of mothers consumed milk and other dairy products.

The proportion of mothers and children who consumed vitamin A-rich fruits and vegetables was 176 (22.3%) and 173 (21.9%), respectively. Besides this, the proportion of other fruits and vegetables consumption in children was 279 (35.3%) and mothers' consumption of other fruit was 245 (31%) and other vegetables consumption was 309 (39.1%) (Table 2).

The median dietary diversity score of mothers is 4, that is, less than the optimum minimum dietary diversity score recommended by the FANTA/FAO-MDD-W, which is ≥5 food groups, and the median dietary diversity of the children is 3, which is also below the optimum minimum dietary diversity score recommended by the WHO-IYCF, i.e., ≥4 food groups.

**Figure 1:**
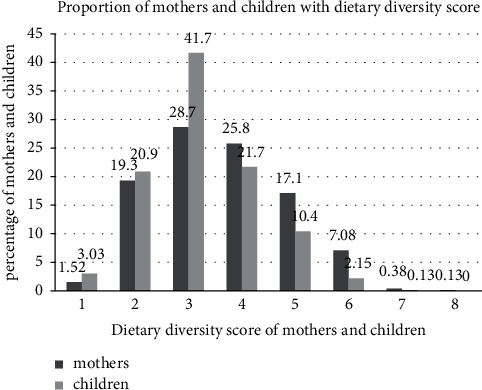
Proportion of mothers and children (6–23 months) with their dietary diversity score in Kucha District, Gamo Zone, South Ethiopia, 2017.

Only one-quarter 195 (24.7%) of mothers consumed ≥5 food groups and also one-third 272 (34.4%) of the children consumed ≥4 food groups, who met the optimum dietary diversity score. The highest proportion of mothers 227 (28.7%) and children 330 (41.7%) consumed only 3 food groups in the previous 24 hours (Figure 1).

### 3.3. Positive- or Negative-Deviant Mothers in Dietary Diversity Consumption

The study showed that 189 (23.9%) mother-child (6–23 months) pairs were discordants. Of these, 133 (16.8%) mothers were positive deviants who buffer their children and 56 (7.1%) mothers were negative deviants. The remaining 463 (58.5%) and 272 (34.4%) were concordant with low dietary diversity and with higher dietary diversity score, respectively, according to the MDD-W criteria of FANTA/FAO for mothers and the WHO criteria of mean dietary diversity criteria (IYCF) for children (Table 3).

### 3.4. The Strength of Concordance in Mother-Child Dietary Diversity

Majority of the rural mothers/children dyads 560 (78%) were concordants, while 42 (57.5%) urban mothers/children dyads were concordants. Kappa statistics on the strength of agreement between the two output variables maternal dietary diversity (≥5/<5 food items from the ten food groups) and child dietary diversity (≥4/<4 from the 7 food groups) showed a moderate concordance in dietary diversity between mother-child dyads (Cohen's Kappa coefficient = 0.43, *P* < 0.001).

### 3.5. Factors Affecting Maternal and Child Dietary Diversity Concordance

Bivariate logistic regression analysis was performed between the following explanatory variables and the outcome variable: maternal age, residence, educational status, being head of the household, main occupation, family size, primary source of food, production of vegetable, rearing milking cow, chicken rearing, fasting animal source foods, meal frequency of mothers and children, receiving antenatal care and postnatal care, receiving dietary advice for the last child, maternal infection, age of the child, sex of the child, place of delivery, child vaccination, growth monitoring and promotion, food refusal of children, child infection, food security and wealth status of the household, dietary diversity score of mothers, and dietary diversity score of children.

Variables with the *P* value < 0.25 in the bivariate analysis were selected as candidates for multivariable logistic regression. Hence, after adjusting for those explanatory variables in the final model, mothers and children who reside in rural area, mothers who had no formal education, households without milking cow, and children with low dietary diversity were found to be positively associated and low maternal dietary diversity was negatively associated with mother-child dietary diversity concordance (Table 4).

Place of residence was found to be a strong factor affecting mother-child dietary diversity concordance. The odds of being concordant were 3.5 times higher for mother-child pairs from rural areas compared with their urban counterparts (AOR = 3.49; 95% CI: 1.91–6.41). Women with no formal education had 1.8 times higher odds of being concordant with their children compared with those who attained secondary and above level of education (AOR = 1.80; 95% CI: 1.08–3.05). Presence of milking cow in the household was found to be a significant factor for mother-to-child dietary diversity concordance. Mother-child pairs who did not own milking cow in the household had 1.7 times higher odds of being concordant compared with those who own milking cow in their households (AOR = 1.7; 95% CI: 1.10–2.56). Mother and child dietary diversity also showed a significant positive and inverse association, respectively, with concordance of their dietary diversity. Children who fed low diversity foods had about 8 times higher odds of being concordant to their mothers compared with those consumed high diversity diets (AOR = 8.23; 95% CI: 5.17–13.08). Mothers who consumed low dietary diversity had 0.46 times lower odds of being concordant to their children compared with those consumed high dietary diversity (AOR = 0.46; 95% CI: 0.29–0.74) (Table 4).

## 4. Discussion

This study examined the concordance between maternal and child dietary diversity and factors affecting the concordance. The study showed that the proportion of discordant is few, which is 23.9% of the total, 7.1% of mothers and 16.8% of children only. Even though the Ethiopian government implemented health extension programs to educate the community on different health packages including maternal and child feeding practices [34], the minimum dietary diversity was 24.7% and 34.4% in mothers and children, respectively. Even though it calls for further efforts in food security and raising of awareness on the importance of dietary diversity, it is higher than the national figure, which is at 10.8% [32], and the nearby district in Gamo Zone, which is 23.3%, in children aged 6–23 months [21]. The dominant dietary food groups consumed were grains, roots, and tubers, which is 99.4% in mothers and 95.8% in children, followed by legumes, pulses, and nuts consumption of 70.5% in mothers and 67.8% in children. Consumption of flesh foods is very low at 4.6% in both mothers and children. The possible explanation for this low consumption of flesh foods might be because of the abundance of starchy foods as stable [35] and the widely common belief that young children are not able to digest flesh foods. Given flesh foods are costy in the current local market, the majority of women from low socio-economic class might not also afford to purchase foods of animal origin. This findings generally have an implication that enhanced efforts are needed in raising wareness on the benefits of diversified diet.

The study also showed that there is agreement between maternal and child dietary diversity as it was revealed in the Kappa statistics (*kappa* = 0.43, *P* < 0.001), which indicates that there is moderate concordance between mother-child dyads in dietary diversity. This is to mean that the more food groups the mothers consumed, the more likely their children achieved their minimum dietary diversity and vice versa. As the mothers' dietary diversity increased, the percentage of children (6–23 months) meeting this criterion increased dramatically. An increase in the percentage of children reaching the minimum dietary diversity was greater with each successive increase in maternal dietary diversity. Even though there is a dearth of literature on concordance between maternal and child dietary diversity, a related study on maternal and child dietary diversity associations in Bangladesh, Vietnam, and Ethiopia showed a fair association between the two [36]. The variation could be attributable to differences in methodology (the current study was conducted using the seven food groups for children and the ten food groups for mothers, while the previous studies used the seven food groups for mothers and children, study settings, study population dynamics, timing of the study, and other related factors). Since majority of women and children have a low DD in the study, the concordance is more of a low DD concordance.

This study also found the odds of being concordant was higher for mother-child pairs from rural areas compared with their urban counterparts. However, of these rural dweller concordants, 77% mother-child pairs did not achieve their minimum dietary diversity score. This showed most rural mothers did not achieve the recommended minimum dietary diversity and also failed to meet their children's. However, 76.2% of the urban dweller concordants achieved the minimum dietary diversity. This result was slightly higher than the study conducted on dietary diversity of Nigerian rural women [37], but some evidence showed that people who reside in rural areas were more likely to adopt their traditional food and fed more diverse foods [38, 39]. However, the reverse was true in this study area as the diets were not varied enough. This low dietary diversity of the rural women could be a function of low socioeconomic status of rural women and low awareness on the importance of diversified diet. Because most of them earn low income, and this may lead them to inability to afford for food varieties. The low dietary diversity score of the rural mothers and children indicates that they may not meet their micronutrient requirements [2, 40, 41].

The study also revealed that maternal education is a significant predictor of maternal-child dietary diversity concordance where mothers having no formal education had higher odds of being concordant with their children compared with those who attained secondary and above level education. The finding contrasts with the fact that maternal education enhances diversity both in the mother and child diets [36, 40, 42]. This could be explained by the situation of Ethiopian mothers where majority of uneducated mothers are housewives who could have a better caring opportunity for their children and might feed from the same pot. Interventions targeting such women could improve the micronutrient deficiency among children. However, the association between higher education and better dietary diversity concordance was reflected by the difference in proportion among educated and uneducated mothers in the study. That is, 85.6% of mothers with no formal education did not achieve their minimum dietary diversity. On the other side, 41% of mothers who attained secondary and above level of education achieved the minimum DD for themselves and their children than the 14.4% who achieved the minimum dietary diversity among women with no education. This result coincides with the study conducted in Bangladesh and Vietnam [36]. Similarly, a study from Zambia on dietary diversity at six months of age also showed that maternal education was positively associated with dietary diversity score [40, 42]. This could be due to maternal knowledge that mothers who were educated take much care of their children and may consume for themselves and feed their children diversified diet compared with those who had no schooling. This suggests that education has positive impact on improving maternal and child DD as educated women are more likely to receive nutrition education, which in turn increases the chance of consumption of diversified diet [42, 43].

The study also showed absence of milking cow was positively associated with maternal and child dietary diversity concordance. Of the concordant mother-child dyads, 52.7% of mothers who own milking cow achieved the minimum DD for themselves and their children. This shows that the proportion of mothers who own milking cow and achieved minimum dietary diversity were higher than those mothers who did not own. This result goes in line with a related study on dietary diversity, feeding practice, and determinants among children 6–23 months in South Ethiopia, which showed that mothers who had access to cow milk fed diversified diet two times more than those who had no access [21]. This association implies that availability of a source of food in the household may influence food intake. Evidence suggests that increased availability of fruits, vegetables, and snack foods in the home was associated with increased intake of each food among preschool age children [13]. Similarly, availability of milking cow in households leads to high consumption of milk by mothers and children that may enhance their dietary diversity.

Moreover, compared with those children who consumed high dietary diversity, those who consumed low dietary diversity had higher odds of being concordant to their respective mothers. The result simply showed that the proportion of children who did not achieve the minimum dietary diversity but being concordant with their mothers was high.

On the other hand, mothers who consumed high dietary diversity had lower odds of being concordant with their children than those mothers who consumed low dietary diversity. The possible reason for this is due to high proportions of high number of discordant children who achieved the minimum DDS and low number of discordant mothers who achieved the minimum DDS. The results were consistent with the existing literature depicting the association of maternal and child diets among preschools and school-aged children and among under 24-month children [13]. The finding was inconsistent with findings from a study conducted in Cambodia, Ghana, and Haiti DHS data where mothers' dietary diversity predicted an increase in child diet in some food groups [5]. The more food groups the mothers consumed, the more likely their child attained the minimum DD and the more they become concordant to each other. However, the finding is consistent with a study from Bangladesh, Vietnam, and Ethiopia [36].

In this study, 16.8% of mothers were positive deviants who buffer their children, and possibly these mothers may have benefited children's diets. These mothers reduced their own consumption to act as a buffer against low food diversity for their children to protect children from low diversity or imbalance of micronutrient deficiency. In general, this study showed that mothers with higher DD have children with higher DD and mothers with lower dietary diversity have children with lower dietary diversity. This suggests that irrespective of children's breastfeeding status, they consume the same food groups as their mothers. Because maternal DD is strongly associated with child DD, diverse diet should be promoted for both mothers and children during the entire span of the first 1000 days of mothers and children.

This study used primary data and was conducted as community-based research and believed to be representative for similar settings that should be considered the strength of this study. However, the study has few limitations to consider. Though DDS has been validated as a useful tool to assess the likelihood of meeting micronutrient requirements, the maternal and children's diet was analyzed only qualitatively as quantity was not taken into account. The study also did not show the strength of association for each food group as only the general food group concordance was shown and it did not consider seasonal variation in DDS. Even though probing technique was used, recall bias could be introduced. Because of low count or proportion of high concordance, factors affecting low concordance were determined. Therefore, caution should be exercised in the interpretation of the findings.

## 5. Conclusions

The study showed that there was a moderate concordance between maternal and child dietary diversity. However, majority of mothers and children did not meet the minimum dietary diversity in the study area as the majorities were low DD concordants. In general, the moderate agreement between maternal-child dietary diversity in this study implies that promoting maternal consumption of a variety of foods could improve the DD of their children too. Being a rural resident, having no formal education, not owning milking cow, and children with low dietary diversity were factors associated with mother-child dietary diversity concordance.

Hence, focusing typically on educating rural mothers and community-based education on the importance and ways of improving both maternal and child dietary diversity especially of the rural women using health extension workers may improve their dietary diversity score and in turn the low concordance. In the long run, capacitating the ownership of milking cows to rural women could also contribute to diversification of both women and children's diet by supplying animal source food. A further longitudinal research is needed to strengthen the findings of this study. To sum up, collaborative action is needed from the health, education, and agricultural sectors to realize dietary diversity among women and children.

## Figures and Tables

**Table 1:** Sociodemographic and economic characteristics of mothers in Kucha District, Gamo Gofa Zone, Southern Ethiopia, 2017.

Characteristics	Category	Number	(%)
Age in years	15–19	39	4.9
	20–24	273	34.5
	25–34	389	49.2
	35–49	90	11.4
Ethnicity	Gamo	685	86.6
	Gofa	48	6.1
	Wolayta	25	3.2
	Amhara	31	3.9
	Others	2	0.3
Educational status	No formal education	362	45.8
	Primary education	237	30
	Secondary and above	192	21.5
Religion	Orthodox	240	30.3
	Protestant	548	69.3
	Others	3	0.4
Residence	Rural	718	90.8
	Urban	73	9.2
Marital status	Married	720	91
	Single	26	3.3
	Divorced	18	2.3
	Widowed	27	3.4
Main occupation	Housewife	546	69
	Student	91	11.5
	Employee	25	3.2
	Daily laborer	31	3.9
	Merchant	65	8.2
	Others	33	4.2
Family size	1–3	385	48.7
	>4	406	51.3
Head of household (mothers)	Yes	82	10.4
	No	709	89.6
Main source of food	Own production (farming)	589	74.5
	Purchasing	163	20.6
	Others	39	4.9
Planting vegetables in backyard	Yes	96	12.1
	No	695	87.9
Presence of milking cow	Yes	278	35.1
	No	513	64.9
Presence of chickens laying eggs	Yes	364	46
	No	427	54
Food security status	Mildly food insecure	83	10.5
	Moderately food insecure	385	48.7
	Severely food insecure	11	1.4
	Food secure	312	39.4
Socio economic status	Poor (low)	297	37.5
	Medium	237	30
	High (rich)	257	32.5
ANC	Yes	557	70.4
	No	234	29.6
Delivery site	Health facility	471	59.5
	Home	320	40.5
PNC	Yes	557	70.4
	No	234	29.6
Diversified diet advice	Yes	554	70
	No	237	30
Maternal febrile illness in the previous 24 hours	Yes	100	12.6
	No	691	87.4
Maternal DDS	≥5 food groups	195	24.7
	<5 food groups	596	75.3
Child DDS	**≥**4 food groups	272	34.4
	<4 food groups	519	65.6

**Table 2:** Proportion of food groups consumption of mothers and children in the previous 24 hours in Kucha District, Gamo Zone, South Ethiopia, 2017.

10 food groups in MDD-W	*n* (%)	7 food groups in IYCF MDD	*n* (%)
1. Grains, white roots and tubers, and plantains	786 (99.4)	1. Grains, roots, and tubers	758 (95.8)
2. Pulses (beans, peas, and lentils)	558 (70.5)	2. Legumes and nuts	536 (67.8)
3. Nuts and seeds	163 (20.6)		
4. Dairy	159 (20.1)	3. Dairy products	568 (71.8)
5. Meat, poultry, and fish	36 (4.6)	4. Flesh foods (meat, fish, poultry, and liver/organ meats)	36 (4.6)
6. Eggs	70 (8.8)	5. Eggs	148 (18.7)
7. Dark green leafy vegetables	353 (44.6)	6. Vitamin A-rich fruits and vegetables	173 (21.9)
8. Other vitamin A-rich fruits and vegetables	176 (22.3)		
9. Other vegetables	309 (39.1)	7. Other fruits and vegetables	279 (35.3)
10. Other fruits	245 (31)		

**Table 3:** The proportion of positive- and negative-deviant mothers among the discordants in DDS in Kucha District, Gamo Zone, South Ethiopia, March 2017.

		DDS of children		Total
		Low (≤3)	High (≥4)	
DDS of mothers	Low (≤4)	463 (58.5%)	133 (16.8%)	596 (75.3%)
	High (≥5)	56 (7.1%)	139 (24.7%)	195 (24.7%)
Total		519 (65.6%)	272 (34.4%)	791 (100%)

**Table 4:** Factors affecting maternal-child dietary diversity concordance in Kucha District, Gamo Zone, South Ethiopia, 2017.

Variables		DDS concordance (*n* = 791)		COR, 95% CI	AOR, 95% CI	*P* value
		Concordant	Discordant			
Residence	Urban	560 (70.8)	158 (28)	1	1	1
	Rural	42 (5.3)	31 (3.9)	2.62 (1.59–4.29)	3.49 (1.91–6.42)	0.001^∗∗^
Education	No formal education	291 (36.8)	71 (9)	1.86 (1.25, 2.78)	1.82 (1.08, 3.05)	0.024^∗∗^
	Primary education	179 (22.6)	58 (7.3)	1.403 (0.92, 2.15)	1.592 (0.95, 2.66)	0.077
	Secondary and above	132 (17.6)	60 (7.7)	1	1	1
Antenatal follow-up	Yes	403 (50.9)	154 (19.5)	1	1	1
	No	199 (25.2)	35 (4.4)	2.17 (1.45, 3.26)	1.494 (0.94, 2.38)	0.092
Presence of milking cow	Yes	167 (21.1)	111 (14)	1	1	1
	No	435 (55)	78 (9.9)	3.71 (2.64, 5.21)	1.68 (1.11–2.56)	0.016^∗∗^
Dietary diversity of mothers	High	463 (58.5)	133 (16.8)	1	1	1
	Low	139 (17.6)	56 (7.1)	1.41 (0.97, 2.02)	0.46 (0.29, 0.74)	0.001^∗∗^
Dietary diversity of children	High	463 (58.5)	56 (7.1)	1	1	1
	Low	139 (17.6)	133 (16.8)	7.91 (5.49, 11.39)	8.23 (5.17, 13.08)	0.001^∗∗^

^*∗∗*^ *P* value significant at 0.05.

## Data Availability

The data sets used and/or analyzed during the current study are available from the corresponding author on reasonable request.

## List of Abbreviations

ANC: Antenatal care
ASF: Animal source foods
DASH: Dietary Approaches to Stop Hypertension
DD: Dietary diversity
DHS: Demographic and Health Survey
EDHS: Ethiopian Demographic and Health Survey
FANTA: Food and Nutrition Technical Assistance project
FAO: Food and Agriculture Organization of the United Nations
HFIAS: Household Food Insecurity Access Scale
IYCF: Infant and young child feeding
MDD: Minimum dietary diversity
MDD-W: Minimum dietary diversity of women (15–49 years)
NGO: Nongovernmental Organization
PNC: Postnatal care
PPS: Probability proportional to size
SNNPR: Southern Nations, Nationalities, and People's Region
SPSS: Statistical Package for the Social Sciences
SRS: Simple random sampling
USA: United States of America
USAID: United States Agency for International Development
UNICEF: United Nations Children's (Emergency) Fund
WHO: World Health Organization.
